# Neonatal autonomic regulation as a predictor of autism symptoms in very preterm infants

**DOI:** 10.1038/s41372-024-01942-2

**Published:** 2024-03-29

**Authors:** Jessica Bradshaw, Christian O’Reilly, Kayla C. Everhart, Elizabeth Dixon, Amy Vinyard, Abbas Tavakoli, Robin B. Dail

**Affiliations:** 1 Department of Psychology, University of South Carolina, Columbia, SC, USA.; 2 Carolina Autism and Neurodevelopment Research Center, University of South Carolina, Columbia, SC, USA.; 3 Department of Computer Science and Engineering, University of South Carolina, Columbia, SC, USA.; 4 Artificial Intelligence Institute, University of South Carolina, Columbia, SC, USA.; 5 College of Nursing, University of South Carolina, Columbia, SC, USA.

## INTRODUCTION

Infants born preterm are at a significantly higher likelihood of having autism spectrum disorder (ASD), with reports of a 10-fold increase in the rate of ASD for very preterm infants (VPT, born <32 weeks gestation) [1] and a 20-fold increase for extremely preterm infants (born <28 weeks gestation) [2]. Yet, the etiological link between preterm birth and ASD remains unknown. Preterm birth and ASD are both associated with neurological differences, notably autonomic nervous system (ANS) dysfunction. ANS dysfunction can be measured using abnormal heart rate characteristics (HRCs) and thermal gradients (TGs), and previous research has linked ANS dysfunction to neurodevelopmental impairment and autism-specific outcomes. As such, ANS dysfunction is a promising early biomarker and potential pathway to ASD, particularly in VPT infants. ASD biomarkers for VPTs would enable earlier access to tailored interventions that improve outcomes for this vulnerable population. The current study describes preliminary findings from a prospective, longitudinal trial of VPT infants with continuous measures of autonomic dysregulation in the first month of life, comprehensive neurodevelopmental monitoring, and ASD follow-up at age 1 year.

## METHODS

A subset of VPT infants from a large, multisite clinical trial [3] were enrolled in this study at birth (*N* = 20). Given previous studies linking ASD to abnormal HRCs and to provide a multimodal measurement of ANS dysfunction, continuous measures of minute-by-minute TGs, defined by the difference between central and peripheral temperatures [3], and hour-by-hour abnormal heart rate characteristics (HRCs), measured using HeRO scores [4], were collected from birth-28 days (>40,000 samples/infant). HeRO scores are generated by a HeRO monitor (Medical Predictive Science Corp), calculated from a proprietary algorithm that considers abnormality of three HRCs: standard deviation of inter-beat intervals, heart rate skewness or asymmetry, and entropy (see Supplementary Information). ANS dysfunction is indicated by abnormal HRCs (HeRO scores >1) or elevated negative TGs (peripheral > central body temperature) [5]. Following NICU discharge, standardized measures of cognition, language, and motor skills were collected at corrected ages 6, 9, and 12 months (see Supplementary Information). At 12 months, assessments of social communication and direct observation of early ASD symptoms (Systematic Observation of Red Flags; SORF) were administered [6-8] (see Supplementary Information). The SORF has been used with infants (9–24 months) with complex developmental and ASD profiles. Informed consent was completed with the mother prior to any study procedures, which were approved by hospital and university institutional review boards. Univariate, multivariate, and robust regression models were used to evaluate neonatal predictors of ASD outcomes.

## RESULTS

**Fig. 1:**
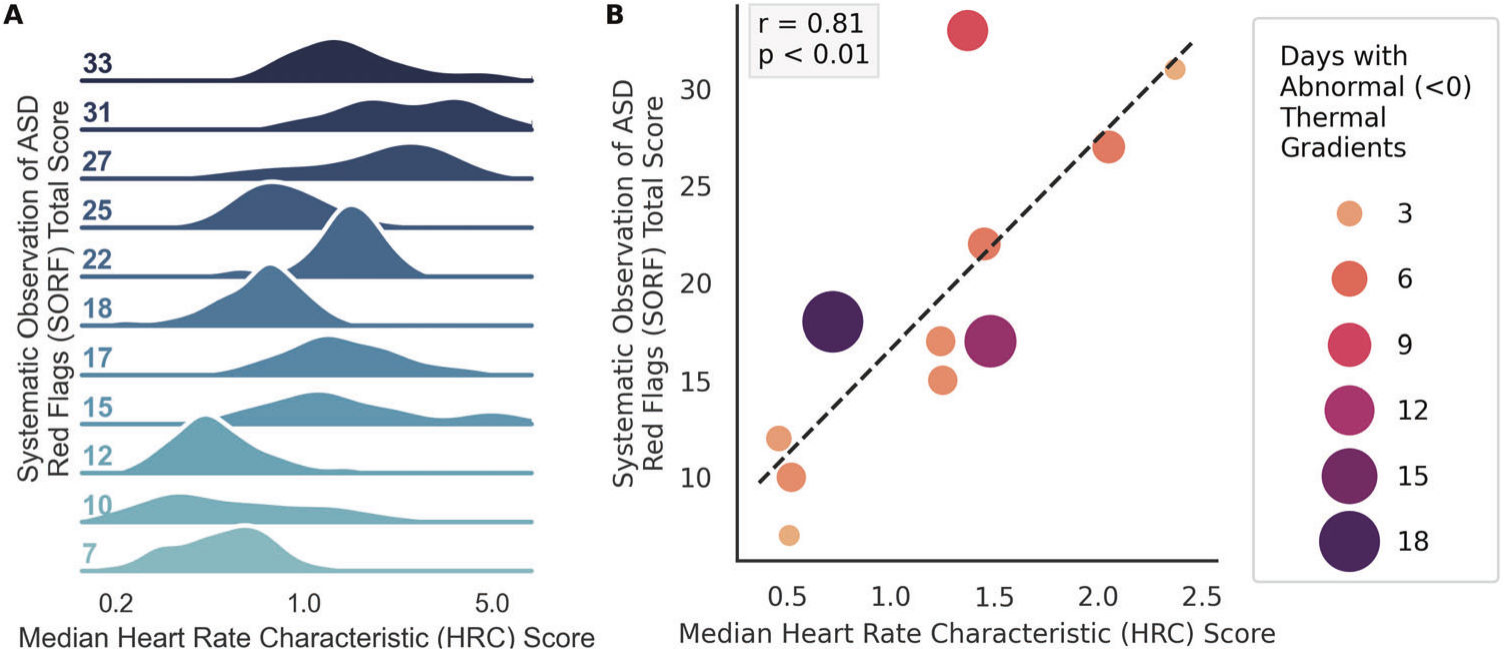
Association between neonatal abnormal heart rate characteristics (HRCs) and 1-year ASD symptoms. Higher scores indicate more HRC abnormality and more ASD symptoms. **A** Ridgeline plot displaying the distribution of neonatal abnormal HRCs (HeRO scores) across participant ASD symptoms (SORF total score) at 1 year. **B** Scatterplot and associated regression between neonatal abnormal HRCs (HeRO scores) across 1-year ASD symptoms (SORF total score).

Descriptive statistics for all measures are presented in Table 1 and Supplementary Fig. S1. Continuous measures of ANS function were collected for *N* = 20 infants enrolled at birth. Following NICU discharge, *n* = 12 completed 1-year developmental follow-up (10 males) and *n* = 1 was excluded from analyses due to significant medical morbidities that precluded a valid ASD assessment (see Supplementary Fig. S1). Across all infants in the first 28 days of life, the average HeRO score was just above 1 and infants experienced abnormal negative TGs for about 20% of the time (6 of 28 days). In terms of developmental trajectories, significant delays did not emerge until 12 months when over 50% of the sample exhibited social-communication delays and exceeded the clinical cutoff for ASD risk (see Supplementary Table S1 and Supplementary Fig S2). Neonatal abnormal HRCs were strongly associated with ASD symptoms at 12 months (*r* = 0.81, *p* < 0.01; Fig. 1), as was birth gestational age (GA), birth weight (BW), and abnormal negative TGs. A regression model including GA, BW, and abnormal HRCs revealed a significant, unique predictive effect of abnormal HRCs on ASD outcomes (model *r* = 0.86, *p* = 0.017; HRC *r* = 0.72, *p* = 0.029).

## DISCUSSION

This report represents the first prospective, longitudinal study to describe predictive associations between neonatal ANS dysfunction, developmental trajectories across the first year of life, and emerging ASD symptoms at 1 year in VPT infants. The clinical presentation for most infants reflected marked delays in social communication that did not emerge until 12 months corrected age. Significant ASD concerns were present for half of the sample by 12 months. ANS measures collected in the first month of neonatal life, more than a year prior to the ASD evaluation, were surprisingly strong predictors of ASD. Existing research points to ANS dysfunction in ASD that can be characterized by an imbalance – sympathetic overactivation and/or parasympathetic underactivation [9, 10]. The strong association between ASD features and abnormal HRCs and, albeit to a lesser extent, abnormal TGs, represents novel evidence contributing to an emerging theoretical model in which ANS imbalance in very early infancy may be an underlying etiological feature of ASD, particularly in VPT infants. Future studies should extend these preliminary findings by including infants born >32w GA, ANS measures that extend beyond the neonatal period, ASD outcomes at later ages, and larger sample sizes. In particular, the prevalence of ASD features reported here should be interpreted with caution, given limited research on the SORF in young VPTs and the complex clinical presentation of VPT infants that includes broad developmental delays. Overall, these results highlight complementary ANS measures that describe how ANS dysfunction, likely resulting from an imbalance between the parasympathetic and sympathetic systems, may impact very early regulatory processes for neonates who later develop ASD. This finding offers a promising avenue for researching etiological mechanisms and biomarkers of ASD.

## Supplementary Material

Supplementary Information

## Figures and Tables

**Table 1.** Descriptive statistics and correlations between neonatal measures and ASD symptoms.

	Mean (SD)	% Atypical^a^	Association with ASD Outcome^b^	*p*-value
*Neonatal measures*				
**Gestational age (weeks)**	**28.25 (2.14)**	**–**	−**0.689**	**<0.0001**
**Birthweight (g)**	**943.3 (227.49)**	**–**	−**0.499**	**0.0031**
Medical comorbidity score^c^	1.08 (0.99)	–	0.241	0.474
Abnormal cranial ultrasound	–	36%	0.031	0.861
**Abnormal heart rate characteristics (HeRO score)**^d^	**1.22 (0.63)**	**–**	**0.812**	**0.002**
**Abnormal negative thermal gradients (days)**^e^	**5.83 (1.18)**	**–**	**0.099**	**0.039** ^f^
*1-year developmental outcomes* ^g^				
Mullen scales of early learning				
Visual reception	48.75 (11.39)	8%	−0.332	0.318
**Fine motor**	**50.01 (15.74)**	**27%**	−**0.661**	**0.037**
Gross motor	40.75 (11.99)	60%	0.028	0.935
Receptive language	34.82 (7.76)	64%	−0.543	0.105
Expressive language	36.5 (12.46)	58%	−0.385	0.243
Communication and symbolic behavior scales – behavior sample (CSBS-BS)				
**CSBS-BS total score**	**74.27 (10.12)**	**72%**	−**0.732**	**0.016**
*1-year ASD outcomes*				
First year inventory (FYI)				
Total FYI risk score	16.95 (7.13)	50%	–	–
Systematic observation of red flags for ASD (SORF)				
Total score	19.00 (8.17)	45%	–	–
Number of red flags	7.18 (2.99)	64%	–	–

Statistically significant correlations with ASD Outcomes are denoted with bold text.

a % Atypical is defined as the percent of infants scoring below average on developmental outcomes and scoring in the “at-risk” range on the ASD measures.

b The primary ASD outcome in regression models is the Systematic Observation of ASD Red Flags (SORF)^7^ Total Score. This score is based on DSM-5 criteria for ASD, comprising both social interaction deficits and restricted interests and repetitive behaviors. Test statistics reported are Wald Chi-Square for categorical predictors (presence of abnormal cranial ultrasound) and Pearson’s *r* for continuous predictors (all other variables).

c Data on medical comorbidities was abstracted from the medical record and consists of the summative total of medical conditions experienced in the NICU (see Supplementary Informationg).

d Abnormal heart rate characteristics are measured using HeRO scores. HeRO scores are calculated with a mathematical algorithm that quantifies abnormality using three components of heart activity: standard deviation of inter-heartbeat (RR) intervals; sample asymmetry; sample entropy.

e Number of days during which negative thermal gradients were present ≥30% of the day.

f Robust regression model used due to outliers in the predictor variable. Robust regression uses estimation techniques to de-emphasize outliers; p-value reported from robust regression model.

g The Mullen Scales of Early Learning was to measure developmental skills at corrected age 12 months. Scores were corrected for prematurity and are reported as t-scores (mean of 50, standard deviation of 10). The CSBS-BS Total Score reflects social communication and symbolic play skills and is reported as a standard score (mean of 100, standard deviation of 15).

## Data Availability

Data is available upon request.
